# ZFP36 inhibits the pro‐apoptotic effects of transforming growth factor β1 on mitral valve interstitial cells via the GTP‐binding protein 6 pathway in mitral valve prolapse

**DOI:** 10.1113/EP092930

**Published:** 2025-11-10

**Authors:** Meng Zhao, Zhaoyi Zhu, Yawei Dai, Li Jiang, Qihan Wen, Yingjie Zhang, Qingyang Shi, Yihu Tang, Jingxin Zhou, Yanhu Wu

**Affiliations:** ^1^ Department of Cardiovascular Surgery First Affiliated Hospital with Nanjing Medical University Nanjing Jiangsu China; ^2^ Department of Cardiology First Affiliated Hospital with Nanjing Medical University Nanjing Jiangsu China; ^3^ Zhongshan School of Medicine Sun Yat‐sen University Guangzhou Guangdong China

**Keywords:** apoptosis, mitral valve prolapse, RNA binding protein, TGF‐β1, ZFP36

## Abstract

The objective of this work was to investigate the role of ZFP36 in mitral valve prolapse (MVP). Mitral valve and plasma were collected to assess the expression of ZFP36, transforming growth factor β (TGF‐β), collagen and elastin and apoptosis rates. Mitral valve interstitial cells (MICs) were transfected with ZFP36 plasmids to observe changes in the secretion of collagen, elastin and matrix metalloproteinases (MMPs) and apoptosis rates. MICs were exposed to TGF‐β1 to evaluate the changes in expression of collagen, elastin, MMPs and ZFP36 and apoptosis. Subsequently, after transfection with ZFP36 plasmid, exogenous TGF‐β1 was added to the MICs, and the secretion of collagen, elastin and MMPs and apoptosis rate were re‐evaluated. Finally, transcriptome and RNA immunoprecipitation (RIP) sequencing was conducted to identify downstream genes of TGF‐β1 that could bind to ZFP36. Patients with MVP showed elevated levels of TGF‐β1 in plasma and increased rates of apoptosis, along with higher expression of ZFP36, TGF‐β1, collagen and elastin in the prolapsed valve. Overexpression of ZFP36 in MICs did not significantly alter the secretion of collagen or elastin or apoptosis rates. TGF‐β1 promoted apoptosis of MICs, increased the secretion of collagen, elastin, MMP‐3,9, ZFP36 and reduced the expression of MMP‐1,2,13. Moreover, overexpression of ZFP36 inhibited the effects of TGF‐β1 on MICs. Co‐analysis of transcriptome and RIP sequencing identified three genes: CFAP184, GTP binding protein 6 (GBP6) and HERC6. Knockdown of GBP6 reduced the pro‐apoptotic effects of TGF‐β1 on MICs. ZFP36 exerts a protective role in MVP by inhibiting the effects of TGF‐β1 on MICs. Notably, ZFP36 can mitigate the pro‐apoptotic effects of TGF‐β1 on MICs through the GBP6 pathway.

## Abstract (highlights)

- **What is the central question of this study?**
  What is the pathogenesis of mitral valve prolapse (MVP)?
- **What is the main finding and its importance?**
  Deposition of collagen and elastin, along with cell apoptosis, are significant pathological phenotypes of MVP. ZFP36 potentially plays a protective role in MVP by mitigating the effects of transforming growth factor β (TGF‐β) on mitral valve interstitial cells (MICs). By binding directly to its mRNA and promoting degradation, ZFP36 can suppress GTP binding protein 6 expression, thus reducing TGF‐β1‐induced apoptosis in MICs, suggesting a possible therapeutic target for MVP.

## 1 INTRODUCTION

In recent years, the prevalence of mitral valve prolapse (MVP) has increased. With an incidence ranging from 0.6% to 3.4% in the adult general population (Melamed et al., 2025), MVP can lead to severe complications such as heart failure, atrial fibrillation and even sudden cardiac death (Constant Dit Beaufils et al., 2021). Mitral chordae tendineae rupture, the most common manifestation of MVP, accounts for 47.5% of cases according to echocardiographic examinations (Wang et al., 2025). A higher frequency of undiagnosed chordae tendineae rupture has been observed during surgical interventions (Yu et al., 2013). Yet, the aetiology of MVP remains poorly understood.

To date, no effective medical treatment has been established to delay the progression of MVP. Hence, surgical intervention remains the primary treatment. In recent years, mitral valvuloplasty has begun to replace mitral valve replacement as the main surgical approach. However, mitral valvuloplasty is limited by the recurrence of mitral regurgitation, often necessitating secondary surgery (David, 2015). Therefore, exploring the aetiology and mechanisms of MVP is essential to address these limitations.

Research on the aetiology of MVP has been sparse. In previous work, ZFP36 was identified as a potential key player in MVP (Zhao et al., 2023). However, the specific roles and mechanisms of ZFP36 in MVP are still unclear. As an RNA‐binding protein, ZFP36 regulates the alternative splicing of pre‐mRNA through its RNA recognition regions (Castello et al., 2012), and exerts regulatory functions by binding to other proteins (Peng et al., 2025). Additionally, ZFP36 acts as an anti‐inflammatory regulator, controlling various immune responses by suppressing the production of pro‐inflammatory cytokines (Makita et al., 2021). Previous studies have highlighted the role of transforming growth factor β (TGF‐β) activation and its associated signalling pathways in prolapsed valves (Geirsson et al., 2012; Hagler et al., 2013; Rizzo et al., 2015).

In summary, this study seeks to clarify whether ZFP36 influences MVP and if its effects are mediated through modulating TGF‐β signalling in mitral valve interstitial cells (MICs).

## 2 METHODS

### 2.1 Human plasma and mitral valve samples

Patients diagnosed with MVP and admitted to Jiangsu Province Hospital were recruited for the study group. Correspondingly, healthy examinees matched by age and gender were selected for the control group. Inclusion criteria for the study group were: (1) patients were diagnosed with MVP by echocardiography; (2) patients had moderate or severe mitral valve regurgitation and required surgical treatment; and (3) coronary angiography ruled out coronary heart disease. Exclusion criteria for the study group were: (1) patients were diagnosed with other serious valve disorders; (2) patients were diagnosed with atrial fibrillation; and (3) patients exhibited severe symptoms of heart failure. The control group's inclusion criterion was that echocardiography was used to exclude cardiac valve disorders. The control group's exclusion criteria were other cardiac structural diseases and cardiac electrophysiological disorders.

Patient blood samples were collected post‐admission and immediately centrifuged for plasma, which was subsequently assessed for TGF‐β1 concentrations using enzyme‐linked immunosorbent assay (ELISA).

Mitral valves from MVP patients were obtained during mitral valve repair or replacement procedures. Concurrently, mitral valves from heart transplant recipients in our hospital served as control samples. The valves were examined via immunohistochemistry, Masson, elastin, and terminal deoxynucleotidyl transferase dUTP nick end labelling (TUNEL) staining.

### 2.2 Cell isolation and culture

MICs were isolated using collagenase (Type II, Thermo Fisher Scientific, Waltham, MA, USA, 9001‐12‐1) digestion (Qiu et al., 2022) and subsequently cultured in Dulbecco's modified Eagle's medium (DMEM, Biosharp, Anhui, China, BL304A) augmented with 10% fetal bovine serum (FBS, NESRA, Shaghai, China, S711‐001) and 1% penicillin–streptomycin (EpiZyme,Shanghai, China, CB010). Cells at passages 4 were employed in the subsequent experiments.

### 2.3 Immunohistochemistry

Primary antibodies applied in this study included TGF‐β1 (Abcam, Waltham, MA, USA, Ab215715) and ZFP36 (Proteintech, Rosemont, IL, USA, 66938‐1‐lg). Secondary antibodies were horseradish peroxidase‐conjugated anti‐rabbit IgG (Beyotime, Shanghai, China, A0208, 1:50), and goat anti‐rabbit IgG 594 (Thermo Fisher Scientific, A‐11012, 1:500). Valve specimens procured during surgery were initially preserved in pre‐chilled phosphate‐buffered saline, subsequently fixed in 4% v/v paraformaldehyde, and embedded in paraffin wax. The embedded tissues were sectioned at a thickness of 5 µm and baked for 2 h at 60°C. After cooling, slides were dewaxed using xylene and a decreasing ethanol gradient from 100% to 70%. Antigen retrieval and endogenous peroxide blocking were performed in accordance with the manufacturer's instructions (Beyotime, Shanghai, China, P0083, P0100B). Upon blocking with normal goat serum (Beyotime, Shanghai, China, C0265), the tissues were subjected to primary antibody application overnight at 4°C. For immunohistochemistry (IHC), sections were exposed to secondary antibody solution for 1 h at room temperature and reacted with a peroxidase substrate kit for 3,3′‐diaminobenzidine (Beyotime, Shanghai, China, P0202). Images were acquired using a Nikon (Tokyo, Japan) E100 microscope.

### 2.4 Cell‐apoptosis analysis

As previously outlined, cells and tissues were prepared, following which a One Step TUNEL Apoptosis Assay Kit (Beyotime, Shanghai, China, C1086) was employed for the detection of apoptosis. For the visualization of nuclei, 4′,6‐diamidino‐2‐phenylindole (Beyotime, Shanghai, China, C1005) was used as a counterstain for a duration of 10 min. Image acquisition was conducted using a fluorescence microscope system (Olympus (Tokyo, Japan) SlideView VS200). For the purpose of this experiment, 100,000 cells were seeded in 24‐well plates and allowed to culture for 48 h. These cells were then subjected to an Annexin V‐FITC/PI Apoptosis Detection Kit (Vazyme, Nanjing, China, A211‐01) treatment and subsequently analysed using flow cytometry. Each sample, consisting of 1,000,000 cells, was analysed, with three independent samples being tested for every condition. The rate of apoptotic cell death was determined using FlowJo software.

### 2.5 Masson staining

Using a Modified Masson's Trichrome Stain Kit (Solarbio, Beijing, China, G1346), human valves were stained as per the manufacturer's guidelines to calculate the collagen volume fraction. The slides were incubated in Mordant Solution at 37°C overnight, followed by staining with Celestite Blue Solution and Mayer Hematoxylin Solution for 2 min each. After 10‐s differentiation in Acid Differentiation Solution, the sections were treated with ponceau–acid fuchsin solution and phosphmolybic acid solution for 10 min each. The aniline blue solution was then added for 5 min. Following these processes, the tendons were sealed and observed using an Olympus SlideView VS200.

### 2.6 Elastic fibre staining

The Elastic Fiber Stain Kit (Solarbio, Beijing, China, G1596) was utilized for elastic fibre staining, following the guidelines provided by the manufacturer. After treatment with Tanake Oxidizing Solution and bleaching with Tanake Bleach Solution for 5 min each, the slides were stained in Victoria Blue Solution, capped for 24 h, and rinsed with 70% alcohol until the staining solution was fully removed. Subsequently, the tendons were sealed and observed under the Olympus SlideView VS200.

### 2.7 ELISA

To ascertain the TGF‐β1 concentration in the plasma of both groups, a Human TGF‐β1 ELISA kit (Ruixinbio, Shanghai, China, SU‐BN11801) was employed. The blood samples were spun in a centrifuge (75004524, Thermo Fisher Scientific) at 3000 rpm for 10 min. Serum was aliquoted and frozen at −80°C until analysis. The procedures for this experiment were strictly in line with the manufacturer's instructions. Results were obtained with a Multiskan FC microplate reader (51119180ET, Thermo Fisher Scientific).

### 2.8 Western blot

Protein extraction was executed utilizing the Exkine protein extraction kit (Abbkine, Wuhan, China, KTP3007) per the manufacturer's guidelines. Subsequently, the extracted proteins (20 µg) were separated via 10% SDS‐PAGE (EpiZyme, Shanghai, China, PG112) electrophoresis and transferred onto a polyvinylidene difluoride membrane (EpiZyme, Shanghai, China, WJ001). The membranes were blocked with protein‐free rapid blocking buffer (EpiZyme, Shanghai, China, PS108P) for 20 min at room temperature. The proteins of interest were subsequently detected via incubation with primary antibodies at 4°C overnight. The membranes were washed and incubated with corresponding secondary antibodies for 2 h at room temperature.

The primary antibodies employed were as follows: anti‐MMP‐1,2,3,9,13 (ABclonal Technology, Woburn, MA, USA, A1191; Proteintech, 66366‐1‐Ig; Cohesion, Suzhou, China, CPA1749; Proteintech, 10375‐2‐AP and ABclonal, A11755; 1:1000), anti‐β‐tubulin (Abbkine, Wuhan, China, ABP0128; 1:1000), anti‐glyceraldehyde 3‐phosphate dehydrogenase (GAPDH; Abbkine, Wuhan, China, ABL1020; 1:5000), anti‐ZFP36 (Proteintech, 66938‐1‐lg; 1:1000), anti‐Collagen I (Abmart, Shanghai, China, TA7001S; 1:1000), anti‐Elastin (Abmart, Shanghai, China, PK33720S; 1:1000).

The detection process was completed by developing the blots with enhanced chemiluminescence reagent (EpiZyme, Shanghai, China, SQ201) and capturing the results using a Tanon 5200 Multi 4600SF (Shanghai, China).

### 2.9 Cell transfection

For siRNA and plasmid transfection, MICs were plated in six‐well microtitre plates. MICs were transfected with siRNA at a final concentration of 50 nmol/L and plasmid at 1.25 mg/L using Lipofectamine 3000 (Thermo Fisher Scientific) when MICs reached 50% confluence. After 24 h of transfection with Opti‐MEM, the medium was replaced with DMEM containing 10% FBS for 24 h. Then, the transfected cells were used for the following experiment. The used siRNA and plasmid are showed in Supporting information, Table S1.

### 2.10 Total RNA extraction, RNA‐seq library construction and sequencing

RNA‐seq were conducted with assistance from Wuhan IGENEBOOK Biotechnology (Wuhan, China) (www.igenebook.com). Total RNA was extracted using the TRIzol method. All the RNA samples were assessed for their integrity using a Qsep400 instrument (Guangzhou, China). To construct RNA libraries with the VAHTS mRNA‐seq V8 Library Prep Kit for Illumina, 1 µg of total RNA was used (Xu et al., 2019). The procedure included polyA‐selected RNA extraction, RNA fragmentation, random hexamer primed reverse transcription, and 150 nt paired‐end sequencing by Illumina (San Diego, CA, USA) Novaseq 6000.

### 2.11 Bioinformatics analysis of RNA‐seq

The adapter and low‐quality reads were filtered out through cutadapt (version 1.11). Clean reads were mapped to the porcine reference genomes by Hisat2 (version 2.1.0), allowing up to two mismatches (Su et al., 2019). These genes were subjected to alignment against a public protein database: NR (RefSeq non‐redundant proteins). Featurecount (v1.6.0) was used for transcript abundance estimation and normalization of expression values as fragments per kilobase of transcript per million fragments mapped (FPKM) (Li and Dewey, 2011). Differentially expressed genes were identified with edgeR with a filter threshold of false discovery rate (FDR) < 0.05 and |log2 fold change| > 1 (Bakhtiarizadeh et al., 2012). ClusterProfiler (http://www.bioconductor.org/packages/release/ bioc/html/clusterProfiler.html) in the R package (Yu et al., 2012) was employed to perform Gene Ontology (GO, http://geneontology.org/) (Ashburner et al., 2000) and Kyoto Encyclopedia of Genes and Genomes (KEGG; Kanehisa and Goto, 2000; http://www.genome.jp/kegg/) enrichment analysis.

### 2.12 RIP‐sequencing

RIP‐seq wAS conducted with assistance from Wuhan IGENEBOOK Biotechnology. The mitral interstitial cell (MIC) sample was cross‐linked at 400 mj/cm^2^ at 4 °C. Total proteins were then isolated, and 50 µL magnetic beads and 5 µg anti‐ZFP36 antibodies were mixed for 30 min at room temperature. The magnetic beads bound with antibodies were incubated with 900 µL total proteins overnight at 4 °C for affinity purification. The ZFP36 proteins were eluted from magnetic beads for 30 min at 55°C. Input RNA and the RNA extracted from RIP eluate were used to construct respective sequencing libraries. The libraries were constructed with the NEBNext Ultra RNA Library Prep Kit following the manufacturer's instructions (New England Biolabs, Ipswich, MA, USA) and sequenced on an Illumina NovaSeq 6000 system with 2 × 150‐bp paired‐end read mode at Novogene Bioinformatics Institute (Tianjin, China).

### 2.13 Statistical analysis

The data are presented as means ± standard deviation (SD). Group comparison was conducted using GraphPad Prism 8.0 (GraphPad Software, San Diego, CA, USA), with the values of the study group compared with those of the control group. For continuous variable data, the Shapiro–Wilk test was first used to determine whether it conformed to a normal distribution and Levene's test was used to detect the homogeneity of variances in the data. For normally distributed data Student's *t*‐test was used for statistical analysis and the Mann–Whitney *U*‐test was conducted for those data that did not confirm to a normal distribution. In addition, categorical data were analysed using a chi‐square test. A *P*‐value <0.05 was indicative of statistical significance.

## 3 RESULTS

### 3.1 Increased expression of ZFP36, collagen and elastin and cell apoptosis in prolapsed valves

**FIGURE 1:**
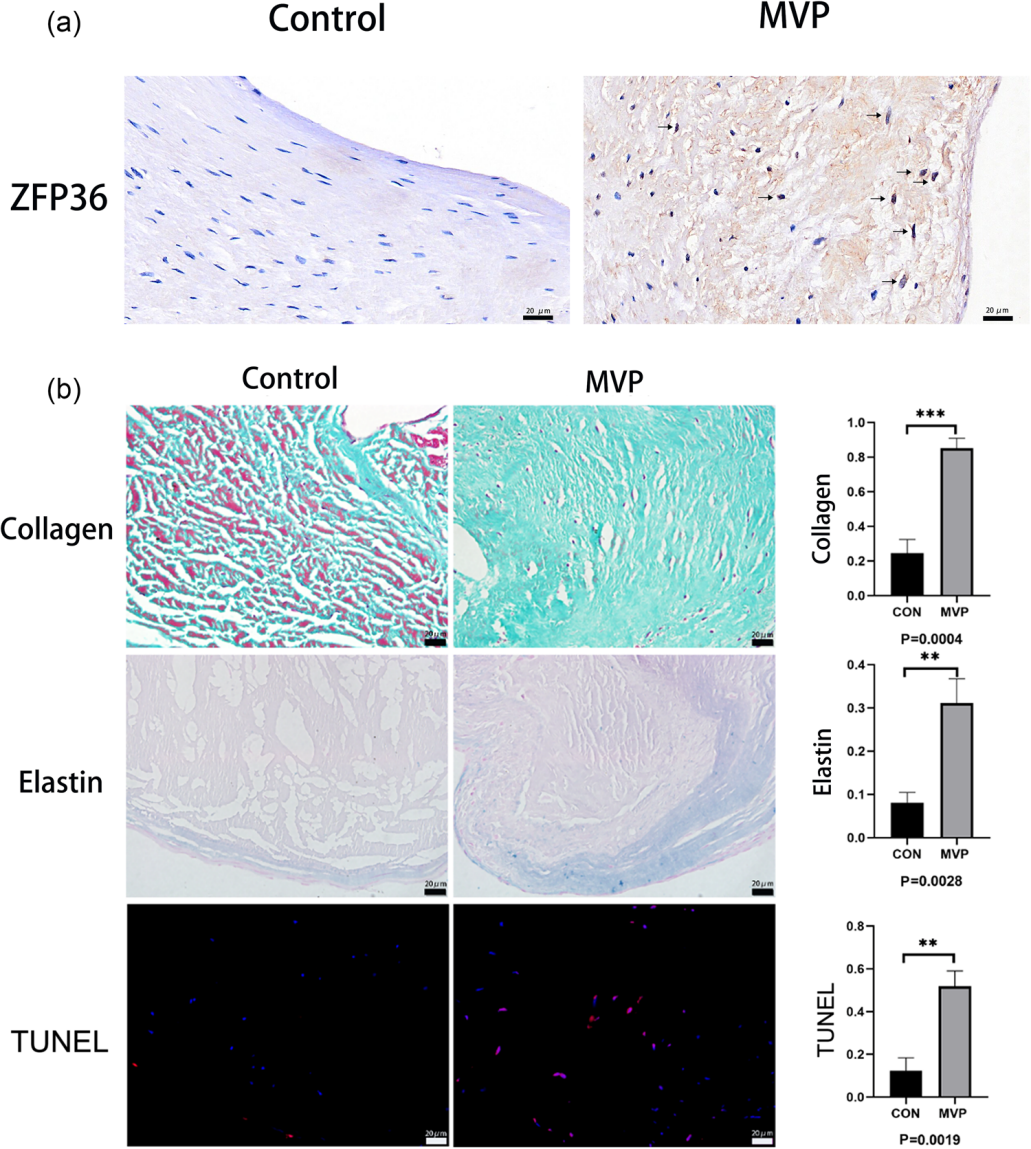
(a) Immunohistochemical staining of ZFP36 in the mitral valve of MVP patients and heart transplant recipients. (b) Masson, elastin and TUNEL staining in the mitral valve of MVP patients and heart transplant recipients. The green part is the collagen fibre and the bule area is the elastin fibre. The data are shown as the means ± SD (Student's *t*‐test, *n* = 5 each in MVP patient group and heart transplant recipient group). **P < 0.01, ***P < 0.001.

**FIGURE 2:**
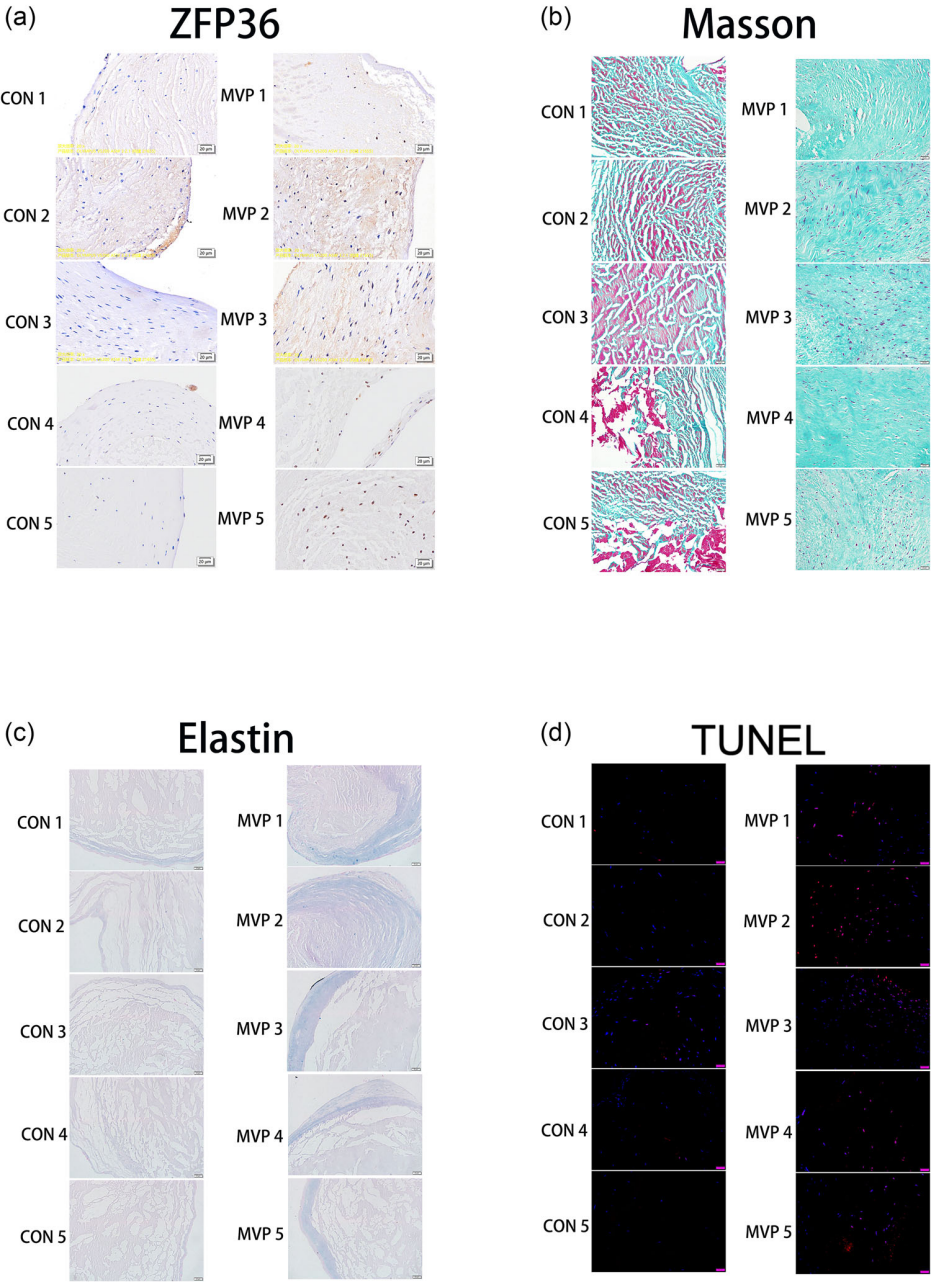
Results of ZFP36 immunohistochemical staining (a), Masson (b), elastin (c), and TUNEL (e) staining.

Three prolapsed mitral valves and three control mitral valves were obtained. Compared with the control group, the expression of ZFP36 was significantly higher in the prolapsed valves, with interstitial cells primarily exhibiting this high expression (indicated by a black arrow in Figure 1a). Furthermore, the expression levels of collagen and elastin fibres were markedly higher in the prolapsed valves (*P *= 0.0004 and *P *= 0.0028, respectively), and the apoptosis rate of cells in these valves was significantly elevated compared to those in control valves (*P *= 0.0019) (Figure 1b). All the results of ZFP36 immunohistochemical staining, Masson, Elastin and TUNEL staining were showed in Figure 2.

### 3.2 Effects of ZFP36 overexpression in MICs

**FIGURE 3:**
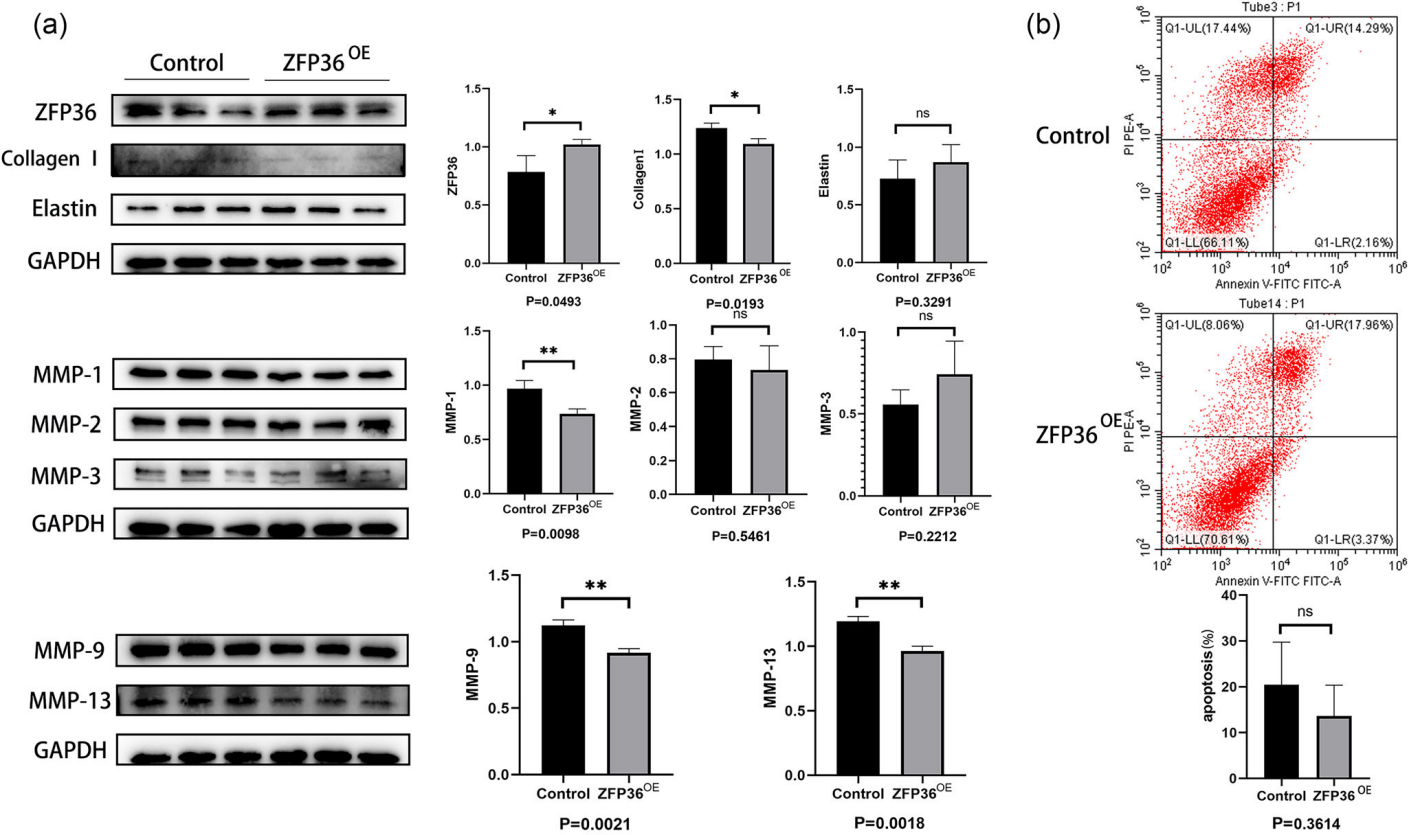
(a) Impact of ZFP36 overexpression on MIC secretion of MMP‐1, 2, 3, 9, 13, collagen I and elastin (*n* = 3 per group). (b) Influence of ZFP36 overexpression on apoptosis (*n* = 3 per group) in MICs. The data are shown as means ± SD, and each experiment was repeated three times. Statistical analysis was done by Student's *t*‐test. *P < 0.05, **P < 0.01.

Following transfection with a ZFP36 overexpression plasmid, MICs showed reduced expression of MMP‐1, 9, 13 and collagen I (*P *= 0.0098, 0.0021, 0.0018 and 0.0193, respectively) compared to the control group. However, overexpression of ZFP36 did not significantly affect the expression of elastin (*P *= 0.3291) or MMP‐2, 3 (*P *= 0.5461, 0.2212) (Figure 3a). Additionally, no significant difference was observed in the apoptosis rate between the ZFP36 overexpression group and the control group (*P *= 0.3614) (Figure 3b).

### 3.3 Increased expression of TGF‐β1 in plasma and valves of MVP patients and its effects on MICs

**FIGURE 4:**
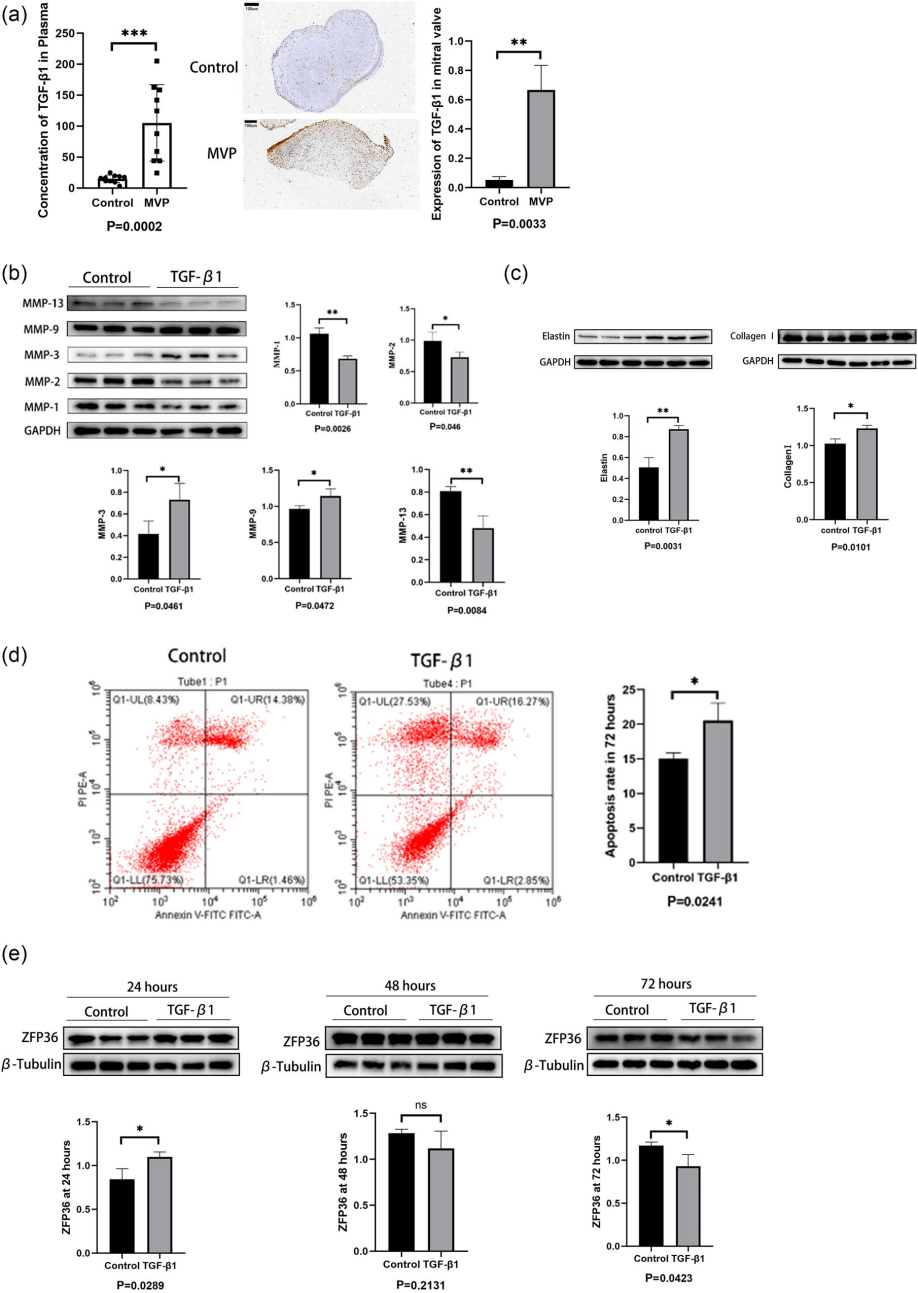
(a) Plasma concentration and mitral valve expression of TGF‐β1 in MVP patients and controls. (b, c) Effects of TGF‐β1 on MIC secretion of MMP‐1, 2, 3, 9, 13 (b), collagen I and elastin (c). (d) Impact of TGF‐β1 on apoptosis in MICs. (e) Expression of ZFP36 after the stimulation of TGF‐β1 for 24, 48, and 72 h. Ten blood samples and 3 valve samples were obtained from MVP group and control group, respectively. The western blot and apoptosis experiments were repeated three times. The data are shown as the means ± SD and statistical analysis was done by Student's *t*‐test. *P < 0.05, **P < 0.01, ***P < 0.001.

Plasma of 10 MVP patients and healthy individuals was obtained, and the basic characteristics of the MVP group and healthy group are shown in Table 1. Relative to the healthy group, the MVP group exhibited a higher concentration of TGF‐β1 in plasma (*P *= 0.0002). Similarly, the expression level of TGF‐β1 was significantly greater in the prolapsed valves than in the control valves (*P *= 0.0033) (Figure 4a). After treatment with 10 ng/mL TGF‐β1 for 72 h, the expression of MMP‐1, 2, 13 was reduced (*P *= 0.0026, 0.046, 0.0084), whereas the expression of MMP‐3, 9 was increased (*P *= 0.0461, 0.0472) (Figure 4b). Concurrently, TGF‐β1 promoted the secretion of collagen (*P *= 0.0101) and elastin (*P *= 0.0031) by the MICs compared with the control group (Figure 4c). Moreover, TGF‐β1 was found to promote apoptosis in MICs (*P *= 0.0241; Figure 4d). TGF‐β1 could promote the expression of ZFP36 at 24 h (*P *= 0.289). However, there was no significant difference between the two groups at 48 h (*P *= 0.2131). Furthermore, the expression of ZFP36 significantly decreased after the stimulation of TGF‐β1 for 72 h (*P *= 0.0423; Figure 4e).

**TABLE 1:** Comparison of basic characteristics between MVP group and healthy group.

Characteristic	Control group (*n* = 10)	MVP group (*n* = 10)	*P*
Age (mean ± SD)	60.4 ± 11.97	63.7 ± 11.02	0.516
Male (*n* (%))	8 (80%)	8 (80%)	—
Weight (kg)	69.6 ± 7.792	68.8 ± 8.574	0.8296
EF (%)	58.2 ± 3.676	59.5 ± 4.478	0.4871
Alcohol history (*n* (%))	2 (20%)	4 (40%)	0.3291
Smoking history (*n* (%))	5 (50%)	4 (40%)	0.6531
Hypertension (*n* (%))	4 (40%)	3 (30%)	0.6392
Diabetes (*n* (%))	0 (0%)	1 (10%)	0.3049
Cancer history (*n* (%))	0 (0%)	0 (0%)	—
Cerebral diseases (*n* (%))	1 (10%)	0 (0%)	0.3049
Digestive diseases (*n* (%))	1 (10%)	1 (10%)	—
Respiratory diseases (*n* (%))	0 (0%)	0 (0%)	—
Renal diseases (*n* (%))	0 (0%)	0 (0%)	—

EF, ejection fraction.

### 3.4 ZFP36 mitigates the effects of TGF‐β1 on MICs

**FIGURE 5:**
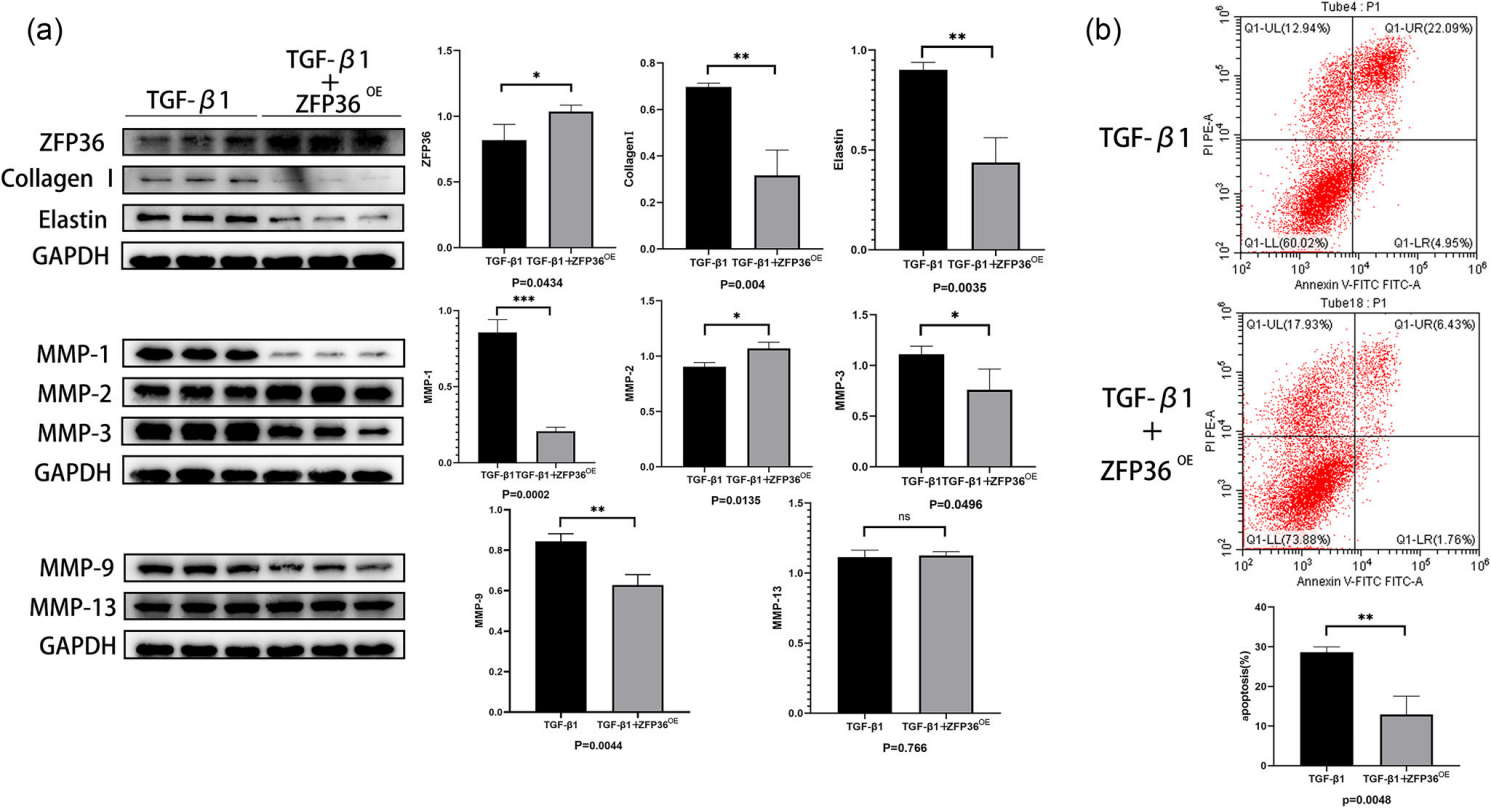
(a) Effects of ZFP36 overexpression on TGF‐β1's influence on MICs regarding the secretion of MMP‐1, 2, 3, 9, 13, collagen I and elastin. (b) Effects of ZFP36 overexpression on TGF‐β1‐induced apoptosis in MICs. The data are shown as the means ± SD, and each experiment was repeated three times. Statistical analysis was done by Student's *t*‐test. *P < 0.05, **P < 0.01, ***P < 0.001.

MICs were divided into two groups: one treated with 10 ng/mL TGF‐β1 (T group) for 72 h and another transfected with a ZFP36 overexpression plasmid followed by treatment with 10 ng/mL TGF‐β1 (ZT group) for the same duration. Compared with the T group, the ZT group exhibited higher expression of MMP‐2 (*P *= 0.0135) and lower expression of collagen I (*P *= 0.004), elastin (*P *= 0.0035) and MMP‐1, 3, 9 (*P *= 0.0002, 0.0496, 0.0044). No significant difference was noted in the expression of MMP‐13 between the two groups (*P *= 0.766; Figure 5a). Additionally, ZFP36 was shown to inhibit the pro‐apoptotic effect of TGF‐β1 on MICs (*P *= 0.0048; Figure 5b).

### 3.5 RNA‐seq revealed differentially expressed genes between T group and ZT group

**FIGURE 6:**
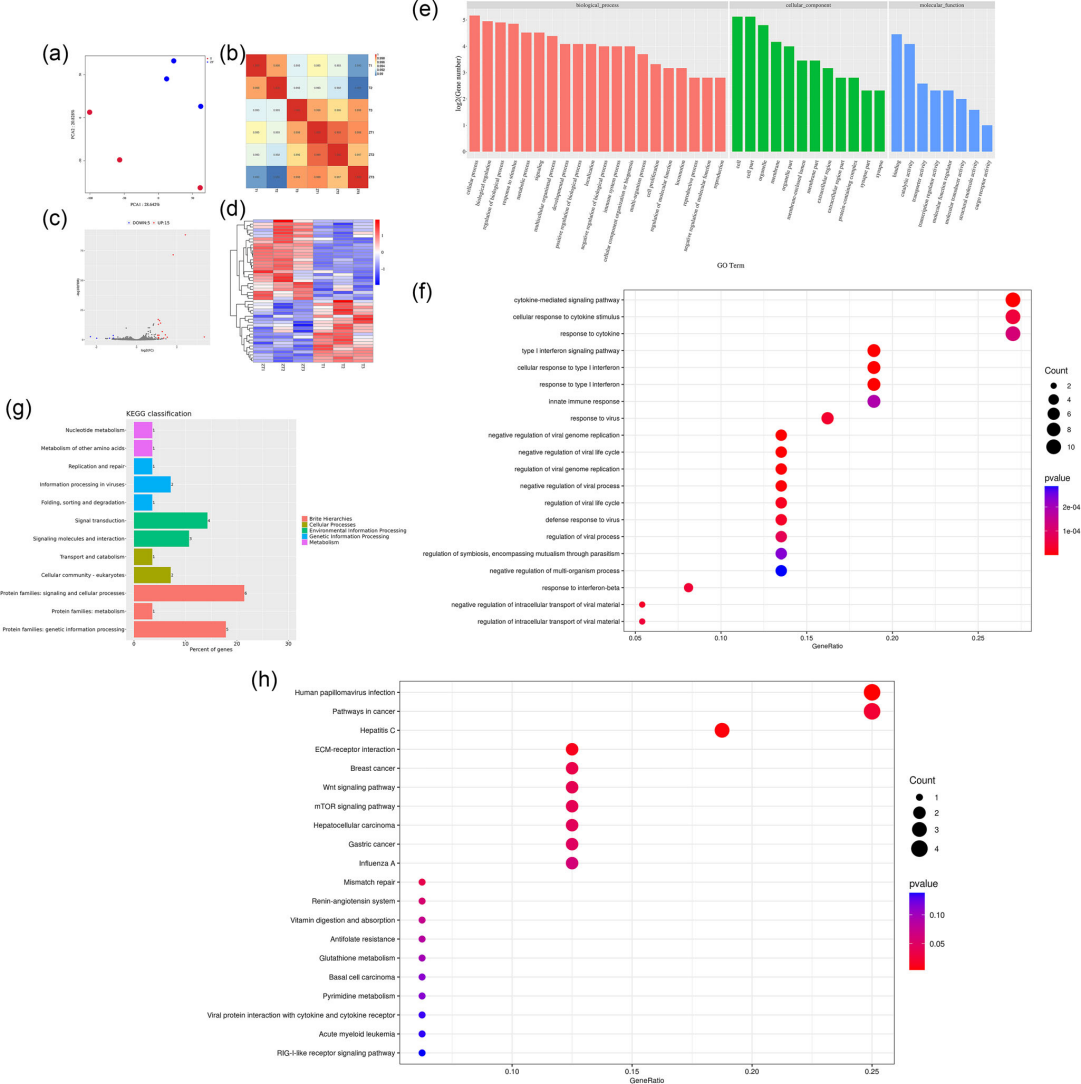
(a) Principal component analysis (PCA) of the T group and ZT group. (b) Inter‐sample clustering analysis of sample correlations between the two groups. (c, d) Volcano plot (c) and heat map (d) of DEGs. (e) GO clustering analysis of all DEGs. (f) GO clustering and enrichment analysis of all DEGs. (g, h) KEGG classification and enrichment analysis of all DEGs. Three samples of T group and ZT group (each samples contained at least 10^6^ MICs) were obtained for RNA sequencing and differentially expressed genes were identified with edgeR with a filter threshold of FDR < 0.05 and |log2 fold change| > 1.

Principal component analysis (PCA) indicated that intra‐group differences were minimal, whereas inter‐group differences were substantial (Figure 6a). Inter‐sample clustering analysis of sample correlation is depicted in Figure 6b. Compared with the T group, the ZT group exhibited 21 downregulated genes and 27 upregulated genes (48 differentially expressed genes (DEGs) are shown in Supporting information, Table S2); the volcano plot and heatmap of these DEGs are presented in Figure 6c and d, respectively. GO clustering analysis of the DEGs revealed the most enriched terms in biological processes as cellular processes, in cellular components as cell and cell parts, and in molecular functions as binding (Figure 6e). The most enriched GO terms included cytokine‐mediated signalling pathways, cellular responses to cytokine stimulus, response to cytokine, type I interferon signalling pathway, and cellular response to type I interferon (Figure 6f). KEGG classification of DEGs showed the most enriched pathway was protein families: signalling and cellular processes (Figure 6g). The most enriched KEGG pathways included human papillomavirus infection, pathways in cancer, hepatitis C, extracellular matrix (ECM)–receptor interaction, and breast cancer (Figure 6h).

### 3.6 RIP‐Seq identified mRNA directly binding to ZFP36

**FIGURE 7:**
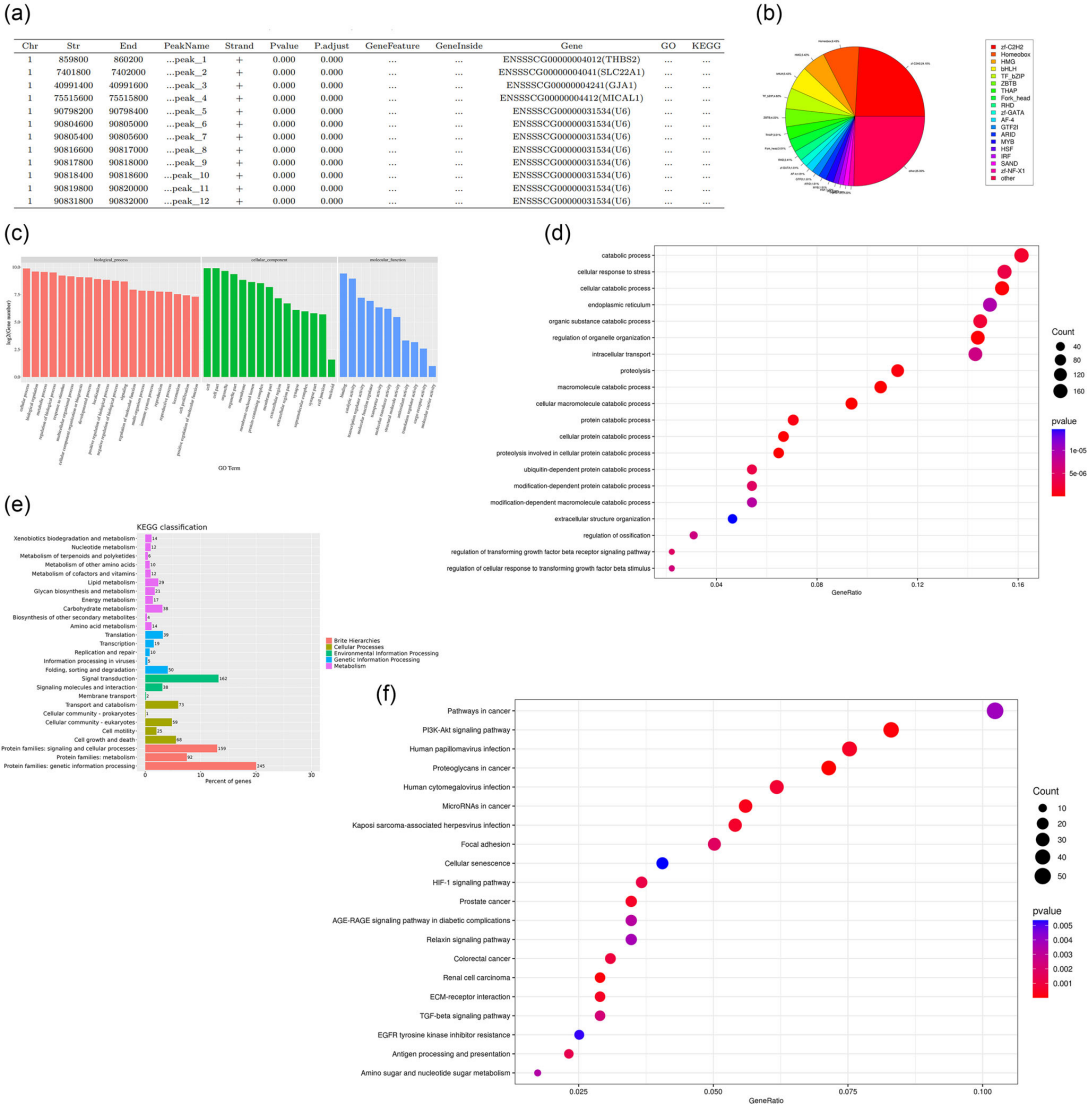
(a) Identification of genes binding to ZFP36 in MICs. (b) Classification of transcription factor families associated with genes binding to ZFP36 in MICs. (c, d) GO clustering and enrichment analysis of genes binding to ZFP36 in MICs. (e, f) KEGG classification and enrichment analysis of genes binding to ZFP36 in MICs. At least 10^7^ MICs were collected for RIP sequencing.

RIP‐sequencing in the ZT group identified mRNAs that bind directly to ZFP36. Of these, 1503 genes were found, and a selection is displayed in Figure 7a (all genes are shown in Supporting information, Table S3). All genes were categorized into 19 transcription factor families, with the highest proportions being zf‐C2H2 (24.1%), Homeobox (8.43%), and HMG (5.42%) (Figure 7b). GO clustering analysis of all genes revealed the most enriched terms were cellular processes in biological processes, cell and cell parts in cellular components, and binding in molecular functions (Figure 7c). The most enriched GO terms were catabolic processes, cellular responses to stress, cellular catabolic processes, endoplasmic reticulum, and organic substance catabolic processes (Figure 7d). KEGG classification of all genes indicated the most enriched pathway was protein families: genetic information processing (Figure 7e). The most enriched KEGG pathways were pathways in cancer, PI3K–Akt signalling pathway, human papillomavirus infection, proteoglycans in cancer, and human cytomegalovirus infection (Figure 7f).

### 3.7 Co‐analysis of RNA‐seq and RIP‐seq

**FIGURE 8:**
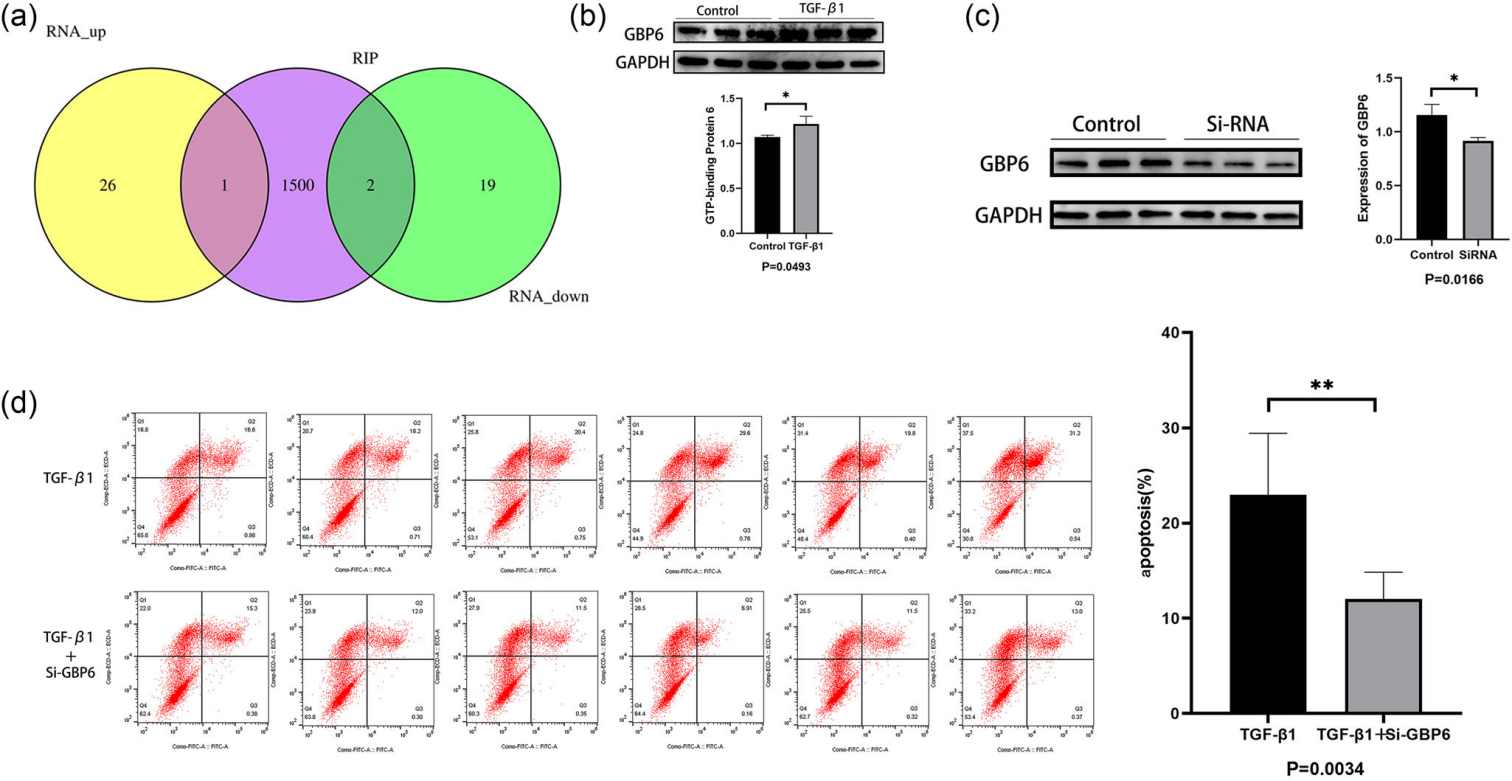
(a) Co‐analysis of RNA and RIP sequencing to identify overlapping genes. (b) TGF‐β1 promoted the expression of GBP6. (c) After transfection with Si‐RNA, the expression of GBP6 significantly decreased (*n* = 3 per group). (d) Effects of GBP6 knockdown on TGF‐β1‐induced apoptosis in MICs (*n* = 6 per group). The data are shown as the means ± SD, and apoptosis experiment was repeated six times. Statistical analysis was done by student's *t*‐test. *P < 0.05, **P < 0.01.

Co‐analysis identified three overlapping genes between RNA sequencing and RIP sequencing: CFAP184, GTP‐binding protein 6, and HERC6. Among these, HERC6 was upregulated, while CFAP184 and GTP‐binding protein 6 were downregulated in the ZT group (Figure 8a).

### 3.8 TGF‐β1 promoted the expression of GBP6 and knockdown of GBP6 mitigated the pro‐apoptotic effect of TGF‐β1 on MICs

Compared with the control group, TGF‐β1 promoted the expression of GTP binding protein 6 (GBP6) significantly (*P *= 0.0493) (Figure 8b). Then, MICs were divided into two groups: one treated with 10 ng/mL TGF‐β1 for 72 h and another transfected with GBP6 siRNA followed by the same TGF‐β1 treatment. Flow cytometry analysis demonstrated that knockdown of GBP6 (Figure 8c) could inhibit the apoptosis induced by TGF‐β1 on MICs (Figure 8d).

## 4 DISCUSSION

Recently, the prevalence of MVP has increased, yet its pathogenesis remains elusive, with few studies addressing it. In prior research, it was demonstrated that ZFP36 might be crucial in MVP. Additionally, associations were suggested between the development of MVP and both the extracellular matrix and cell apoptosis (Zhao et al., 2023). Consequently, this study examined prolapsed valves and noted elevated ZFP36 expression, as well as increased collagen fibre and elastin levels, alongside a higher apoptosis rate in these valves. Similar findings were reported by Geirsson et al. (2012) regarding the expression of col1A1, col3A1 and elastin in affected valves, aligning with our results. In conclusion, it is hypothesized that accumulations of collagen and elastin and cell apoptosis are significant pathological features of MVP. Furthermore, Yang et al. (2024) identified that MICs play a central role in secreting MMPs and in the degradation and remodelling of the extracellular matrix. Thus, the changes in the extracellular matrix caused by MICs are an important pathological feature of MVP.

Initially, ZFP36‐overexpressing MICs were created, revealing that mere overexpression of ZFP36 did not markedly influence the secretion of MMP‐2, 3 and elastin or apoptosis. As a cytoplasmic protein (Zhang et al., 2020), ZFP36 is extensively expressed in the heart, lungs and liver, binding to and destabilizing target mRNAs. Known targets include TNF, IL6, IL8, IL17A, IL33, VEGFA, HIF1A and MMP9 (Makita et al., 2021). ZFP36's involvement in various autoimmune conditions such as rheumatoid arthritis and psoriasis has been verified (Son et al., 2019). The research of Son et al. (2019) suggested that ZFP36L1 aids in osteoarthritis by enhancing ECM degradation. However, since ECM accumulation characterizes MVP, the facilitative role of ZFP36 in ECM degradation may be beneficial, positing ZFP36 as an anti‐inflammatory agent and TGF‐β as a known pathogenetic factor in MVP. Therefore, it is proposed that ZFP36 could mitigate the effects of TGF‐β on MICs, thereby exerting a protective function in MVP.

TGF‐β has three subtypes: TGF‐β1, TGF‐β2 and TGF‐β3. Elevated levels of all three subtypes were observed, but only the TGF‐β1 receptor was found to be increased in prolapsed valves (Geirsson et al., 2012). Consequently, TGF‐β1 was selected for further investigation. It was discovered that TGF‐β1 was significantly upregulated not only in the plasma but also in the prolapsed valves of patients with MVP, consistent with prior findings. In vitro studies showed that TGF‐β1 could enhance the expression of collagen I and elastin and induce apoptosis in MICs, mirroring the pathological features of MVP. In conclusion, TGF‐β1 is the key pathogenic factor for MVP.

To explore the interaction between TGF‐β1 and ZFP36, we treated the MICs with TGF‐β1 for 72 h. However, the expression of ZFP36 was decreased compared with the control group, which was not consistent with the immunohistochemical staining. The study of Blanco et al. (2014) found that TGF‐β could promote the expression of ZFP36 at 24 h. Therefore, we measured the expression of ZFP36 after the stimulation of TGF‐β1 for 24 and 48 h. We found that expression of ZFP36 showed a trend of first increasing and then decreasing under the stimulation of TGF‐β1. Therefore, we hypothesized that TGF‐β1 can promote the expression of ZFP36, and ZFP36 can reflexively inhibit the effect of TGF‐β1 on MICs until ZFP36 is exhausted.

Following this, ZFP36‐overexpressing MICs were developed, and TGF‐β1 was exogenously introduced. It was found that ZFP36 could mitigate the impact of TGF‐β1 on the secretion of MMP‐2, 3, 9, collagen and elastin and apoptosis in MICs, supporting our earlier hypothesis. To elucidate the mechanisms underlying ZFP36's inhibition of TGF‐β1, RNA sequencing was conducted to identify DEGs affected by ZFP36, alongside RIP sequencing to determine the mRNAs that directly bind to ZFP36 in MICs. Joint analysis of both RNA and RIP sequencing data revealed three common genes: CFAP184, GTP‐binding protein 6 and HERC6. Hence, ZFP36 was shown to directly bind to these genes, potentially influencing the degradation or overexpression of their mRNAs to inhibit TGF‐β1's effects on MICs.

To date, the role of CFAP184 remains unexplored, although mutations in related genes such as CFAP47 (Liu et al., 2021), CFAP58 (Sha et al., 2021) and CFAP65 (Li et al., 2020; Wang et al., 2021) have been linked to abnormal sperm flagella development and male infertility. The association between CFAP184 and MVP necessitates further investigation. Previous studies have noted that HERC6 is upregulated in the peripheral blood mononuclear cells of patients with systemic lupus erythematosus, where it induces apoptosis in monocytes and promotes disease progression (Cao et al., 2022). Contrary to these findings, our study revealed a negative correlation between HERC6 expression and cell apoptosis, indicating a need for additional research to clarify this discrepancy. GBP6, part of the GTP‐binding protein family, has been documented to promote apoptosis in lung cancer cells (Choi et al., 2009) and macrophages (Wang et al., 2022), while also showing potential inhibitory effects on apoptosis in T lymphocytes (Moorman et al., 1996) and neuronal cells (Bazenet et al., 1998). So, we explored the association between GBP6 and apoptosis of MICs. We found that TGF‐β1 could facilitate the expression of GBP6 and upon knocking down GBP6 in MICs using siRNA, we observed a reduction in TGF‐β1‐induced apoptosis, aligning with the findings of Wang et al. (2022). So, our study proved that ZFP36 can inhibit the pro‐apoptotic effects of TGF‐β1 by directly binding to GBP6's mRNA and enhancing its degradation.

Our study, based on clinical samples and cell experiments, confirmed the protective effect of ZFP36 in MVP. This provides a potential therapeutic target for the treatment of MVP. However, this study still has some shortcomings. First, the clinical sample size was limited because of the rarity of MVP and the wide application of mitral valve reconstruction. Verification with a larger sample size is needed in the future. Second, the study mainly focused on the study of phenotypes, and the research on the mechanism was not deep enough. Third, due to the lack of a model of MVP in animals, this study lacks animal‐based experimental research.

### 4.1 Conclusion

ZFP36 is protective in the development of MVP, attenuating the impact of TGF‐β1 on MICs by reducing the secretion of MMP‐2, 3, 9, collagen and elastin and apoptosis. Specifically, ZFP36 inhibits GBP6 expression by directly binding to its mRNA and enhancing its degradation, thus diminishing TGF‐β1‐induced apoptosis in MICs.

## AUTHOR CONTRIBUTIONS

Yanhu Wu, Jingxin Zhou and Yihu Tang: conceptualization and supervision. Meng Zhao, Zhaoyi Zhu and Yawei Dai: project administration. Li Jiang and Qihan Wen: writing—original draft and writing—review & editing. Yingjie Zhang and Qingyang Shi: software and formal analysis. All authors have read and approved the final version of this manuscript and agree to be accountable for all aspects of the work in ensuring that questions related to the accuracy or integrity of any part of the work are appropriately investigated and resolved. All persons designated as authors qualify for authorship, and all those who qualify for authorship are listed.

## CONFLICT OF INTEREST

The corresponding author and all co‐authors have no conflicts of interest to declare.

## Supporting information



Supplemental Table S1: Sequence of GTP‐binding protein 6 siRNA and ZFP36 plasmid.

Supplemental Table S2: 48 differentially expressed genes between ZT and T group.

Supplemental Table S3: Information of 1503 mRNAs that can bind directly to ZFP36.

## Data Availability

The data that support the findings of this study are available from the corresponding author upon reasonable request.
